# A Library of Old Photos Supporting Conversation of Two Generations Serving Reminiscence Therapy

**DOI:** 10.3389/fpsyg.2021.704236

**Published:** 2021-08-31

**Authors:** Lei Jiang, Panote Siriaraya, Dongeun Choi, Noriaki Kuwahara

**Affiliations:** ^1^Graduate School of Science and Technology, Kyoto Institute of Technology, Kyoto, Japan; ^2^Faculty of Informatics, The University of Fukuchiyama, Kyoto, Japan

**Keywords:** two-generation conversation support, reminiscence therapy, photo types, emotion assessment scales, old photo library

## Abstract

In Japan, a shift in family patterns has led to a sense of social isolation among older people, which increases the risk of major neurocognitive disorder. Interventions for them using old photos to implement reminiscence therapy (RT) have been proved to be effective. A super-aged society has in turn led to a shortage of medical resources and older people prefer home care over institutional care. Therefore, there is an urgent need for volunteers to help in RT. However, the age of volunteers tends to be increasingly younger. The lack of knowledge and experience of the past for the young volunteers makes it difficult for them to select appropriate stimulated materials. To improve this situation, a library of old photos for RT was developed to support conversation between the two generations. A two-factor experiment and emotion assessment scales were designed to explore the effect of different old photo types on the fluency of conversation between the two generations and their emotion. It was found that the types of old photos have little effect on older people and that conversations were almost pleasant. However, the pleasantness of older people was enhanced when using photos that they wanted to talk about (*P* = 0.006). Meanwhile, pleasure in conversation of the older people increased with the attention of the young people to the topic (*R* = 0.304, *p* < 0.001). Conversely, photo type has a strong impact on young people. When photos are selected that older people do not want to talk about or photos that young people do not know the content and are not interested in, concern for the topic of young people drops dramatically. Therefore, when RT, it is important to avoid using the types of photos above that cause a drop in younger people's attention.

## Introduction

### Background

#### Relationship Between Social Isolation and Reminiscence Therapy

The aging rate of Japan has reached its highest level since 2005, and it will continue to increase^1^. In 2018, the population aged 65 or over was 355.08 million, 28.1% of the total population, and this percentage is expected to reach 33.3% in 2036^2^. Adults aged 60 or over, more than 20% suffer from mental or neurological disorders, with the most common mental and neurological disorder being depression and major neurocognitive disorder^3^. In Japan, the shift from traditional extended families (consisting of three or more generations) to nuclear families (consisting of parents and children under 18 years of age) living in a large city with limited space in recent decades has led many older people to live alone and feel socially isolated (Kuiper et al., 2015). Social isolation is an independent risk factor in major neurocognitive disorder, and it also increases the risk of other factors that contribute to major neurocognitive disorder, such as cognitive inactivity, hypertension, and depression (Leroi et al., 2018). At the same time, older people experience stressors in their lives that can lead to feelings of isolation or psychological distress. In short, the cause of major neurocognitive disorder is complex and multifactorial, but the onset of full-blown major neurocognitive disorder can be delayed for several years by intervention using cognitive techniques (Livingston et al., 2017).

One effective intervention is reminiscence therapy (RT), an approach having therapeutic effects for older people both with and without major neurocognitive disorder, which can stimulate the brain and has the effects to slow down the functional decline of the brain and the progression of major neurocognitive disorder (Yamagami et al., 2007). RT is a therapy advocated by American Psychiatrist Robert Butler in 1963 (Butler, 1963). The participants engage in reminiscence activities with older people in a compassionate, affectionate, and supportive manner that will improve depressive symptoms, achieve psychological stability, help older people reassess their lives and strengthen their self-identity, thereby improving their quality of life and interpersonal relationships (Jo and Song, 2015). In Japan, the Memory Center was established in 2002 in Aichi Prefecture, and empirical studies have been conducted in several regions, showing improvements in cognitive function and quality of life among the older people.

#### Nursing Shortage

Reminiscence therapy, however, requires a significant amount of healthcare resources, and the aging society has exacerbated the limited resource of caregivers. Since 2003, various robots such as Paro, QRIO, and Robovie have been developed to serve as communication, interaction, and therapy aids in health care for the older people. However, 34% of caregivers in Japan think the introduction of robots will increase loneliness among the older people. And caregivers in Finland also believe that care robots will not alleviate the anxiety and loneliness of the older people (Coco et al., 2018). Meanwhile, most Japanese older people prefer home care rather than institutional or medical care^2^. As such, there is an urgent need for young volunteers to fill this huge gap in care resources. However, most young volunteers lack the ability to implement RT, and they need professional training in order to communicate with the older people smoothly. Therefore, it is urgent that a solution is found to alleviate this problem.

### Research Proposal

A library of old photos is necessary to develop to cover for the lack of experience of young volunteers in implementing RT and to increase the motivation of the two generations to communicate in RT. At the same time, the library can stimulate the memories of the older people while supporting unburdened and enjoyable communication between the two generations.

### Related Works

There are many studies on communication support system, using multimedia contents and robots for RT, including direct and indirect interaction systems.

#### Direct Interaction Systems

CIRCA is a multimedia system using photos, videos, music, graphics, and text displayed on a touch screen for major neurocognitive disorder patients and caregivers. It provides cognitive support for major neurocognitive disorder patients, including communication, entertainment, and creativity (Astell et al., 2004, 2008, 2010). A personalized RT cognitive training system was designed. It is an Internet-based system allows patients and caregivers to access and use the system from any location. (Sarne-Fleischmann et al., 2011). In another study, a comparison between the stress of using photos and using videos was made when communicating with older people and revealed that videos were less stressful than photos for young volunteers (Iwamoto et al., 2015). However, the conversations between the older people and the young volunteers lasted longer when using photos. Iwamoto et al. (2017) used facial expression analysis to determine whether young volunteers felt frustrated and uncomfortable when talking to the older people and found that the level of uncomfortableness depended on the category of the photos. However, only four photo categories of food, home appliances, and play and festival events were studied. In a later study, a mobile conversation program was designed to provide talking points for young people by collecting and organizing digital media related to the pasts of older people and relatives, serving to bridge conversational gaps and facilitate intergenerational interactions (Welsh et al., 2018). In another study (Imtiaz et al., 2018), a mobile phone program was developed in which caregivers selected important events in the app that affected the lives of the individuals and selected photos, music, or text related to this event as cues to prompt the individuals to recall. Long-term use of the app reduced symptoms of psychological symptoms of dementia (BPSD). In the later study, the authors (Coelho et al., 2020) used virtual reality (VR) headsets to allow people with dementia to watch videos with personal relevance to facilitate recall. It was found that most participants enjoyed themselves and often spontaneously shared memories related to the videos. However, slight eye strain and head swelling occurred with prolonged wear. Another recent study (Fu et al., 2020) explored the effects of an interview with older people in long-term care facilities who recalled nostalgic scents on their physiological and psychological responses. It was found that older people who recalled nostalgic scents (the experimental group) experienced more effective reductions in anxiety and depressive symptoms than the group that participated in daily conversations (the control group). Finally, a dialog system based on a deep learning model has been proposed. It has been designed to automate RT by using a photo of a user as input to generate questions about his or her life (Carós et al., 2020). By installing the system on the smartphone or laptop of one, the therapy can be made available to anyone suffering from major neurocognitive disorder.

There are two limitations of such previous studies, however: one is that the information in the interactive system is generally based on the information of the older adults or relatives and has no generality or lack of diversity. This limits the variety of content in the reminiscence therapy materials and causes a lack of topics for younger volunteers who do not know older people and how to communicate with them. One study (Wu et al., 2020) used the fMRI (functional magnetic resonance imaging, which uses magnetic resonance imaging to measure the hemodynamic changes caused by neuronal activity) technique to compare the effects on the brain of a baseline group with a group with major neurocognitive disorder when using old public photos for RT, demonstrated that, on patients with major neurocognitive disorder, using old public photos has the same effect of stimulating autobiographical memory as old personal photos. Secondly, only a limited variety of public old photos was used. Thus, we need to use a wider variety of old public photos to support conversation between the two generations.

#### Indirect Interaction Systems

In 2003, because of the shortage of caregivers, the geriatric care and treatment robot, Paro, was introduced into a long-term experiment of robot-assisted activities for the older people in health care facilities. It was found that interactions with Paro improved the mood of the older people (Wada et al., 2005). A controlled trial found that Paro significantly reduced loneliness in older adults compared to a toy lion and an alive dog (Takayanagi et al., 2014). In another study, Kuwahara et al. (2006) developed a web-based RT, combining IP video telephony with photo and video sharing tools. Patients with major neurocognitive disorder communicate with their therapists *via* a video call. Their experiment showed that networked reminiscence conversations were generally as effective on major neurocognitive disorder patients as face-to-face. In a later study, a remotely operated, remote-controlled robot called Telenoid was also developed to allow older people living alone or in nursing homes to have more opportunities to communicate with family members or volunteers (Kuwamura et al., 2016). In both face-to-face and Telenoid-mediated communication situations, two of the three AD (Alzheimer's disease) patients with moderate AD were found to respond positively to talking with the Telenoid. In another study, In another study, an SNS (social networking service, such as communication via LINE or Facebook) agent bot system that supports multiple multimedia communication types was designed for interactive communication between older people and young people (Kobayashi et al., 2018). The robot is equipped with a microphone, camera, speaker, projector, and web access. Older people can send text, voice, and video messages to the young people without using their smartphones. Young users can then receive messages and reply in real time through LINE.

However, a recent study found that when treating behavioral and psychological symptoms of major neurocognitive disorder with robot-assisted activity (RAA), the participants with cognitive decline did not prefer to use a communication robot compared with the control group (Goda et al., 2020). They had difficulty understanding how to communicate with the robot and were unable to experience the direct benefits of RAA, but there were still positive effects on both groups. Therefore, in order to conduct effective RAA with this group, it may be useful to choose a method that is better understood by the participants.

In summary, there are many multimedia systems and various robots with interactive functions designed to support RT, most of which have achieved great results as mentioned above—Paro robot, Telenoid robot, etc. However, first, the new technologies are difficult for older people to understand, and it takes time for them to learn how to use new technologies. Second, previous studies have mainly used only the personal materials of older people for RT. Although some studies have used public materials and compared the discomfort caused to young people by different types of materials, the variety of materials is limited. Third, it has not yet been studied whether young people know about or are able to understand the content of the materials used for RT. It also has not been explored whether the state of the young people affect the older people, whether interest in the reminiscence therapy material and the fluency of the conversation of the young persons affect the emotion of older adults during the conversation, nor whether there is a uniform standard to quantify the impact of the materials on the conversation between the two generations. Fourth, a prior study (Airi et al., 2010) indicated that when students were looking for photo or video materials for talking with older people, they found it difficult to come up with an appropriate word in their minds to select the material.

### Present Work

In this paper, we aim to address the following objectives:

1. Explore the types of photos suitable for conversation between the two generations through a two-factor experiment.
2. Quantify the emotions that different types of photos may bring to participants during a conversation between the two generations based on the results of the experimental analysis.
3. Develop a library of old photos: label the photos with themes (trigger words provided for young people when looking for conversation photos) and quantified emotional values (a prediction provided before the conversation).

## Materials and Methods

### Design

Reminiscence therapy can be carried out using old photos to stimulate the older people to recall and talk about their experiences and unforgettable memories of their life. Therefore, the photographs chosen were mainly from the years 1926 to 1989. For the young people, almost all of the photos were unknown, which can cause stress for them during the conversation. For older people, whether they want to talk with the young people or not after seeing the photos would also affect the conversation. Therefore, we designed a two-factor experiment to investigate the main factors influencing the conversation:

Variable 1: Two levels about whether older people want to talk when they see the photo: want to (a1) and do not want to (a2).
Variable 2: Three levels whether the young people know the content of the photo: know (b1), do not know but are interested (b2), and do not know and are not interested (b3).
Independent variables: Combining the levels from variables 1 and 2, the photos were divided into six categories a1b1, a1b2, a1b3, a2b1, a2b2, and a2b3.
Dependent variables: A total of six independent variables, rating on the PAS (for the older people, Q1: pleasure, Q2: arousal, rating −4 to 4; Q3: stress_E, rating 1–7) and NCS (for young people, Q1: nature, Q2: concern, rating −4 to 4; Q3: stress_Y, rating 1–7) emotional assessment scales (details shown in Appendix) filled out by the participants at the end of each photo conversation.

### Materials

A total of 206 photos were collected from the Showa Museum website^4^ and Google images based on four themes: Showa era: 130 photos (consisting of eight topics: tools for living, memories of a hot summer, preparation for a cold winter, raising children, memories of wooden school buildings, toys, a street view, electric appliances, dim sum, eco-friendly tools), landscapes: 35 photos, food: 27 photos and festivals: 14 photos. All the Showa era photos mentioned above are the exhibits in the Showa era exhibition of the museum (1926–1989), which were collected over a long period of time by the Reminiscence Therapy Care Prevention Organization and the Museum of History in Nagoya, Japan. And the photos of the other three themes were taken from previous conversation experiments that made older people pleasant during conversation for RT. These photos have been proved to be effective for RT and were allowed for individual use.

### Participants and Procedure

The older people and the students had no previous experience of participating in reminiscence therapy. They had not received any training prior to the experiment. In order not to limit the content of their conversations, it was only explained to the students in advance to guide older people to reminisce based on the photos. The experiments related to this study were approved by the 116th and 122nd Kyoto Institute of Technology Ethics Committee for Scientific Research Involving Human Subjects (Nos. 2020-18 and 2021-03). After the whole process and purpose of the experiment were explained to all the participants, they obtained their approval and they signed the consent form before the formal experiment.

In the first week, the 206 photos referenced in the materials section were organized into a questionnaire, 16 young people (recruited from Kyoto Institute of Technology, Kyoto, Japan) and 12 older people (recruited through Silver Human Resources Center, Kyoto, Japan; aged over 65 years; without cognitive impairment) were invited to answer the photo questionnaire separately. The question under each photo for older people was as follows:

Do you want to talk to young people about this photo? 1. Yes, 2. No.
The questions under each photo for younger people were as follows:
Do you know the content of the photo? If you do not know, would you be interested in talking to the older people about this photo? 1. know, 2. do not know but interested, 3. do not know and not interested.

However, after analyzing the results of the questionnaires from the 28 participants, it was found that the questionnaire answers showed significant individual differences. Therefore, in the second week, 11 older people and 7 young people (some of whom participated in the conversation experiment two times) from the 1st week were randomly put into pairs and participated in the conversation experiment (Table 1). In addition, some personal information about the older people who participated in the conversation experiment is also shown in Table 1. The 206 photos were divided into two levels (a1 and a2 as elderly_t) according to the answers of the older people. The 206 photos were divided into three levels according to the answers of the young people (b1, b2, and b3 as young_t). Then, each photo was divided into six categories (a1b1, a1b2, a1b3, a2b1, a2b2, and a2b3 as photo_type) by combining the answers of the two participants. Finally, six photos were randomly selected from each category, which made 36 photos for each group of the experiments.

**Table 1:** Participants groups.

**Elderly**	**Age**	**Gender**	**Family size**	**Economic status**	**Forgetting things**	**Young**	**Age**	**Gender**
1	74	M	Couple	Slightly plenty	No	1	21	M
2	69	F	Couple	Plenty	Sometimes	1		
3	69	M	Couple	Slightly tight	Sometimes	2	23	M
4	75	F	With brother	Slightly tight	Sometimes	3	23	F
5	82	M	Couple	Slightly tight	Sometimes	3		
6	74	F	Couple	Plenty	Often	4	20	M
7	66	M	Alone	Slightly tight	No	4		
8	68	M	Couple	Slightly tight	Sometimes	5	24	M
9	73	M	3-Generations	Slightly plenty	Sometimes	6	24	M
10	68	F	Couple	Slightly plenty	Sometimes	7	22	F
11	68	F	With mother	Slightly plenty	Sometimes	7		
Mean (SD)	71.25 (4.66)						22.4 (1.51)	

The older people and young people talked in pairs for 1 min about one photo displayed on a large screen. In order to exclude the influence of the display order of photos in different categories of the conversation, the photos were sorted by the La Square distribution. At the end of each conversation, the older people and the young people filled out the emotion assessment forms named, respectively, PAS and NCS scales (see section Emotion Assessment Forms and Appendix for details) to evaluate the conversation. After all the conversations were completed (36 photos), the older people filled out the experimental evaluation form (see Appendix).

In particular, in order to assess the psychological state of recruited subjects before the conversation experiment as well as to indicate the personality characteristics of the subjects, the following individual assessment forms were completed by the elderly people and the young people before the conversation experiment. The 11 older people completed the Japanese version of GDS Depression Quantification Form (Sugishita and Asada, 2009) and the Japanese version of UCLA Loneliness Quantification Form (Masuda et al., 2012). The scores of GDS in the older people (mean, 2; SD, 1.79; range 0–5) for men (mean, 1.5; SD, 1.87) and for women (mean, 2.6; SD, 1.67). A score of >6 on the GDS scale indicates a tendency toward depression. None of the older people in this experiment showed depressive tendencies. The UCLA scale ranges from 20 to 80, with higher scores indicating greater loneliness. The scores distribution for the UCLA scale in the older people (mean, 35.9; SD, 6.8; range, 27–50) is that the mean(SD) value is 36.83(8.30) for males and the mean(SD) value is 34.8(5.12) for females. The 7 young people completed the Japanese version of BIS/BAS scale (Kamide and Daibo, 2005). The BIS/BAS scale consists of 20 items that participants rate on a four-point scale (1: strongly agree, 2: agree, 3: disagree, 4: strongly disagree). Four final scale scores were generated, one BIS score was used to assess the reactions of the young people in the face of stress, and three BAS scores were used to assess the extroverted personality of young people, all with lower scores being better. Specifically, the BIS is associated positively with negative emotions, responding to behavioral inhibition (mean, 16.57; SD, 1.40; seven items; range, 7–28). The BAS is associated positively with extraversion and positive emotions, including reward responses (mean, 11.86; SD, 1.86; five items; range, 5–20, focusing on positive responses to the occurrence or anticipation of reward), desire motivation (mean, 7.0; SD, 1.73; four items; range, 4–16, focusing on whether the desired goal will be pursued with persistence), novelty seeking (mean, 5.43; SD, 1.51; four items; range, 4–16, focusing on reflecting the desire for new rewards and potential events).

### Emotion Assessment Forms

The pleasure, arousal,stress emotion assessment form (PAS scale see Appendix) completed by the older people at the end of the conversation was modified and designed based on the self-assessment manikin (SAM) form. The SAM form is based on the PAD (pleasure, arousal, dominance) dimensional emotion model, a dimensional emotion quantification method accepted by most researchers, using the image of cartoon characters to represent the values of the three dimensions in the PAD model (Gunes et al., 2011). Pleasure dimension is a measure of the level of pleasure of one, from one extreme (upset) to another (ecstasy); arousal dimension is a measure of the level of physiological activity and mental alertness, such as calmness and boredom for low arousal and excitement for high arousal; dominance dimension is a feeling that the surrounding environment and others are influenced by oneself, high dominance is a powerful sense of dominance, while low dominance is a sense of withdrawal (Guo'an and Yinghong, 2013). Experimental participants need to choose the most suitable for their current state in each dimension, each dimension generally consists of five different images of cartoon figurines, and the interval between each figurine can also be chosen (represents the intermediate state of two cartoon figurines). However, in a previous study of older people using the SAM scale for emotion assessment, it was found that they did not know how to choose the dominance dimension, and that they tended to check the cartoon villain directly and ignore the position between the two cartoon villains. According to Russell (Russell et al., 1989), two dimensions, pleasure and arousal, can represent most of the different emotions. Therefore, only these two dimensions were retained and converted into nine-valued scales with values ranging from −4 to 4, indicating the status of the dimension from not at all to extremely. Taking into account whether or not stress is felt during the conversation between the two generations is also an important factor influencing emotion during reminiscence therapy, the stress dimension was added to the emotion assessment form with values ranging from 1 to 7, indicating no stress at all to extremely.

The nature, concern, stress scale is based on some previous studies (Iwamoto et al., 2017; Xiaochun et al., 2019) and previous surveys of young people, which concluded that the main factors affecting emotions of young people when having a two-generation conversation are whether the conversation can be carried out smoothly and continuously, the level of interest in the topic, and the perceived pressure. Therefore, the NCS scale (see Appendix) was designed to assess emotional state of young people based on these three factors.

### Analysis

Statistical analyses were performed using IBM SPSS Statistics, version 26. First, overall analysis to analyze whether there is distribution differences between the six dependent variable values (the rating of PAS and NCS scales: pleasure, arousal, stress_E, nature, concern, stress_Y) based on the independent variable (six photo types), the non-parametric Kruskal–Wallis test was used. The purpose was to explore whether there was any significant effect of photos with interactions (a1b1, a1b1, a1b3, a2b1, a2b2, a2b3, a3b1, a3b2, and a3b3) on the emotional state (six dependent variable values) of older people and younger people. Then, two groups—the older people and the young people—were analyzed separately for each factor level. The hypothesis was that each group would have the same distribution on a1 and a2 as well as the same distribution on b1, b2, and b3 for each group. A non-parametric independent sample test was used to test these hypotheses. It is to explore the effect of the type of photo without interaction on the conversation emotions of the respective groups. Finally, to explore whether there is a correlation between the assessed values of PAS and NCS scales. Since both are ordered variables, they can be considered as fixed distance variables. Correlation analysis was performed using Mantel–Haenszel chi-square tests and using Pearson's correlation coefficient to indicate the degree of correlation. The purpose was to reduce the number of emotion values that labeled the photo in the old photo library.

## Result and Discussion

### Overall Analysis

As shown in Table 2, the mean value of concern for a2b3 (0.12) is significantly lower than the other photo types. Furthermore, Table 3 (Kruskal–Wallis test) indicates that there is a significant difference in conversation depending on the interest of the young people in the topic (*P* = 0.001). Other assessed values on photo types indicate that there is no significant difference across photo types.

**Table 2:** The PAS and NCS mean (SD) report in photo types.

	**Elderly**			**Young**		
**Group**	**Pleasure**	**Arousal**	**Stress_E**	**Nature**	**Concern**	**Stress_Y**
a1b1	2.33 (1.46)	1.59 (1.47)	1.77 (0.87)	3.15 (1.45)	1.67 (2.49)	1.44 (0.90)
a1b2	2.35 (1.41)	1.39 (1.46)	1.77 (0.89)	3.09 (1.76)	1.94 (2.33)	1.36 (0.94)
a1b3	2.23 (1.39)	1.41 (1.60)	1.86 (0.97)	3.15 (1.53)	0.82 (2.45)	1.50 (1.0)
a2b1	2.12 (1.26)	1.33 (1.34)	1.77 (0.78)	2.79 (1.82)	1.33 (2.49)	1.53 (0.95)
a2b2	1.91 (1.29)	1.29 (1.29)	1.83 (0.95)	2.97 (1.41)	1.00 (2.54)	1.36 (0.62)
**a2b3**	1.94 (1.33)	1.30 (1.32)	1.76 (0.84)	2.97 (1.83)	**0.12 (2.83)**	1.47 (1.03)
Total	2.15 (1.36)	1.39 (1.41)	1.80 (0.88)	3.02 (1.64)	1.15 (2.58)	1.44 (0.91)

*N = 66 for each group, a total in 396. Older people: a1, want to talk; a2, not want to talk; young people: b1, know; b2, do not know, interested; b3, do not know, not interested*.

**Table 3:** Kruskal–Wallis Test (grouping variable: photo type).

	**Pleasure**	**Arousal**	**Stress_E**	**Nature**	**Concern**	**Stress_Y**
H(K)	9.182	1.752	0.301	3.198	20.629	1.845
Df	5	5	5	5	5	5
Sig.	0.102	0.882	0.998	0.670	0.001^**^	0.870

*Null hypothesis: the distribution of each sample is the same across categories of photo type*.

** *p < 0.05, reject the null hypothesis*.

The six groups of photo types are further compared in pairs for their concern values (Table 4). It was found that a2b3 with a1b1 (*P* = 0.014) and a1b2 (*P* = 0.001) pairs had the most significant differences in terms of the concern. Combined with the mean of the concern (Table 2), a1b1 (1.67) and a1b2 (1.94) are high and significantly higher than a2b3 (0.12). From the above results, it was already known that the interaction of a and b factors has an effect on the concern for the topic when young people talk, especially at a2b3 where the effect was the greatest and the mean value of concern was only 0.12.

**Table 4:** Pairwise comparison of photo types in concern.

**Type 1-Type 2**	**Test statistic**	**Std. error**	**Std. test statistic**	**Sig**	**Adj.Sig^a^**
a2b3-a1b3	23.295	19.303	1.207	0.227	1.000
a2b3-a2b2	32.311	19.303	1.674	0.094	1.000
a2b3-a2b1	47.977	19.303	2.486	0.013	0.194
**a2b3-a1b1**	64.008	19.303	3.316	0.001	0.014^**^
**a2b3-a1b2**	75.591	19.303	3.916	0.000	0.001^**^
a1b3-a2b2	−9.015	19.303	−0.467	0.640	1.000
a1b3-a2b1	−24.682	19.303	−1.279	0.201	1.000
a1b3-a1b1	40.712	19.303	2.109	0.035	0.524
a1b3-a1b2	52.295	19.303	2.709	0.007	0.101
a2b2-a2b1	15.667	19.303	0.812	0.417	1.000
a2b2-a1b1	31.697	19.303	1.642	0.101	1.000
a2b2-a1b2	43.280	19.303	2.242	0.025	0.374
a2b1-a1b1	16.030	19.303	0.830	0.406	1.000
a2b1-a1b2	27.614	19.303	1.431	0.153	1.000
a1b1-a1b2	−11.583	19.303	−0.600	0.548	1.000

*Each row tests the null hypothesis that the type 1 and type 2 distributions are the same. Asymptotic significance (two-sided tests) is shown*,

** *p < 0.050. a. Significance values have been adjusted by the Bonferroni correction for multiple tests. Older people: a1, want to talk; a2, not want to talk; young people: b1, know; b2, do not know, interested; b3, do not know, not interested*.

To further explore the distribution of Concern on single-factor level, the following two null hypotheses were tested. The first, the distribution of Concern is the same across categories of Young_t (independent-samples Kruskal–Wallis Test); The second, the distribution of Concern is the same across categories of Elderly_t (independent-samples Mann–Whitney U Test). The results were all rejecting the two null hypotheses (*P* = 0.001 and *P* = 0.013). This indicates that there are differences in the distribution of concern across categories at both the a (elderly_t) and b (young_t) levels. In fact, Concern of the Young_t factor from Table 5, the mean of Concern is larger for b1 (1.5) than b2 (1.47). At the same time b1 and b2 are significantly higher than b3 (0.47). This shows that when young people are not interested in the content of photos, they care significantly less during the conversation. It is clear that concern is significantly different for a1 and a2 (*P* = 0.013; mean, 1.47, 0.82). This indicates that the young people showed more interest in the topic when the older persons wanted to talk compared with the older persons who did not want to talk when they saw the photos.

**Table 5:** The young mean (SD) report in levels a and b.

	**a1**	**a2**	**b1**	**b2**	**b3**	**Total**
Nature	3.13 (1.58)	2.91 (1.69)	2.97 (1.65)	3.03 (1.59)	3.06 (1.68)	3.02 (1.64)
Concern	1.47 (2.46)	0.82 (2.66)	1.5 (2.49)	1.47 (2.47)	0.47 (2.66)	1.15 (2.58)
Stress_Y	1.43 (0.94)	1.45 (0.88)	1.48 (0.92)	1.36 (0.79)	1.48 (1.01)	1.44 (0.91)

*a1, a2, N = 198; b1, b2, b3 = 132; a total in 396. Older people: a1, want to talk; a2, not want to talk; young people: b1, know; b2, do not know, interested; b3, do not know, not interested*.

In summary, not only the type of photo for interaction had an effect on the interest in a topic of a young person—the single factor (a and b) photo types also had an effect on the young people. For the young people, as shown in Tables 2, 5 when a2 (0.82), b3 (0.47), and a2b3 (0.12) types of photos were used during the conversation, it made them care less about the topic.

### Single-Factor Level Analysis

From Tables 2, 3, it can be seen that the interactive photo type (photo_type) did not affect the older people significantly. Therefore, the two factors, elderly_t and young_t, were analyzed to determine whether there was an effect on the conversation for the older people.

**Figure 1:**
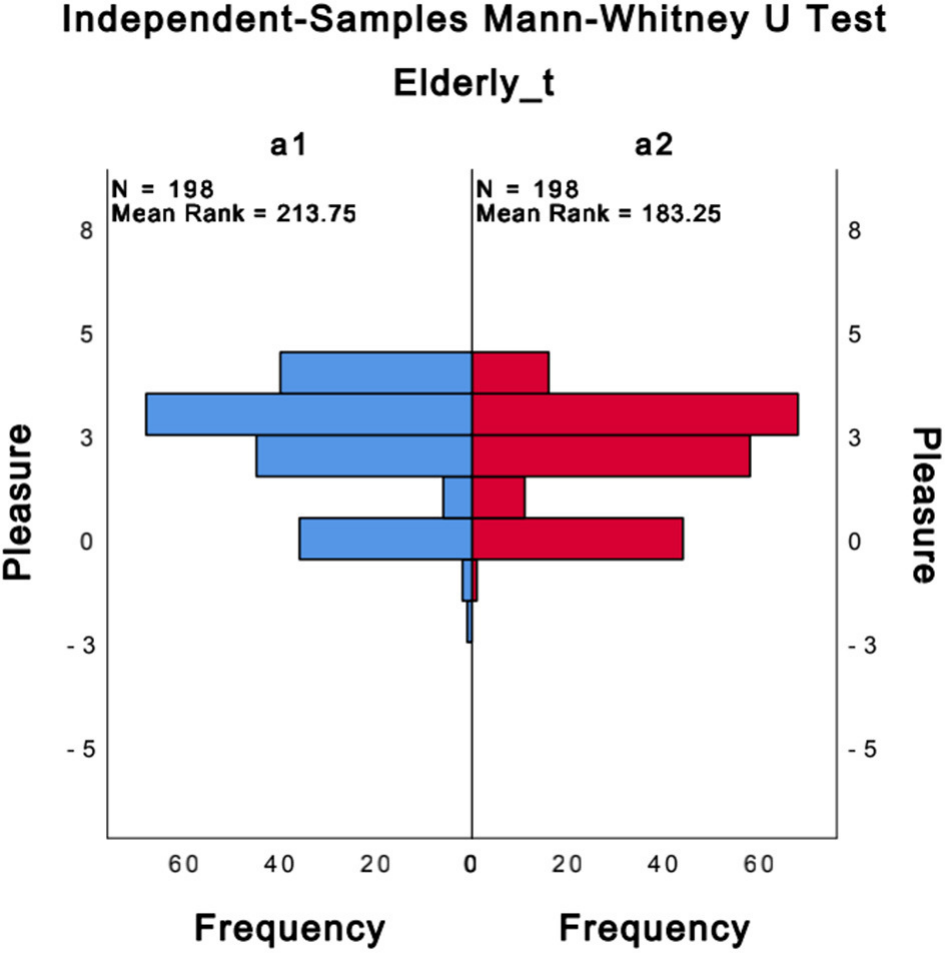
Mann-Whitney U test (Pleasure in a1 and a2).

From Table 6, it is obvious that the mean values of pleasure are only different for a1 (2.3) and a2 (1.99) levels, so it was analyzed further, using the Mann–Whitney U test for level a and Kruskal–Wallis test for level b. The results found the pleasure (*P* = 0.006) distribution was different for level a (elderly_t) (distribution shown in Figure 1). And then, by the same tests found that the arousal and stress_E were not significantly different for levels a and b. In particular, as shown in Figure 1, the number of 4 in pleasure (the highest value of pleasure) for a1 was significantly greater than for a2, which is consistent with the theory presented in the study (Cappeliez and O'Rourke, 2006) that reminiscence can maximize its positive effect by establishing context and providing content, and that recalling positive events from the past enhances the positive effects. However, for both a1 and a2, the number of responses where pleasure was <0 was small, and almost all were >0. And the mean value of a is 2.15 (pleasure, a total of nine intervals from −4 to 4). This suggests that memories brought about by any type of photo have some positive effect on older people. This is in agreement with the theory presented in the study (Cappeliez et al., 2008) that states that narrative-type memories generally create or amplify positive emotions. Pleasure (*P* = 0.623) distribution for the b level does not change significantly.

**Table 6:** The elderly mean (SD) report in levels a and b.

	**a1**	**a2**	**b1**	**b2**	**b3**	**Total**
Pleasure	2.30 (1.41)	1.99 (1.29)	2.23 (1.36)	2.13 (1.36)	2.08 (1.36)	2.15 (1.31)
Arousal	1.46 (1.50)	1.31 (1.31)	1.46 (1.41)	1.34 (1.37)	1.36 (1.46)	1.39 (1.41)
Stress E	1.80 (0.91)	1.79 (0.86)	1.77 (0.82)	1.80 (0.92)	1.81 (0.91)	1.80 (0.88)

*a1, a2, N = 198; b1, b2, and b3 = 132; total in 396. Older people: a1, want to talk; a2, not want to talk; young people: b1, know; b2, do not know, interested; b3, do not know, not interested*.

In summary, there is no significant effect on the b level for the pleasure level during the conversation of the older person. The only significant effect was on the pleasure level of the older people themselves, level a, about whether they wanted to talk or not when they saw the photos. The frequency of 4 for pleasure is greater for a1 than a2. And the mean value of a1 is greater than the mean value of all other levels. Therefore, for the older people, using more photos of type a1 (positive events) for RT can maximize positive emotions in older people.

Overall, the only factor that significantly influenced conversation of the elderly for pleasure in terms of photo was level a. However, it is necessary to further investigate whether the level of concern of young people about the topic (which was also significantly influenced by level a and the interaction ab) had a role in the pleasure of the older people during the conversation. Using Mantel–Haenszel chi-square test between the pleasure variable and the concern variable found that the value of linear-by-linear association was 36.513, and asymptotic significance (two-sided) was *p* <0.001. This only indicated that there was some linear association between the two variables and could not reveal the strength and direction. To determine the strength and direction of the linear association, the Pearson correlation coefficient is required. Therefore, by calculating the Pearson correlation coefficient the result was 0.304 (*P* < 0.001), which indicates a moderate positive association between the concern of the young person about the topic and the pleasure of the older adult in conversation. This indicates the concern of a young person for the topic increases, the pleasure for the older person in the conversation increases. Therefore, combined with the results of section Overall Analysis, this indicates that, by avoiding the use of three types of photos, b3 (mean, 0.47), a2 (mean, 0.82), and a2b3 (mean, 0.12), which make the concern level of the young person increase, the pleasure of the older person will increase.

### Correlation Analysis Between Emotion Evaluation Values

The Mantel–Haenzel chi-square test and the Pearson correlation coefficient were used sequentially to verify the correlation and to determine the strength and direction of linear association between the variables in the two assessment scales (PAS and CNS). It discovered that pleasure is strongly positively correlated with arousal (*P* < 0.001, *R* = 0.662) and weakly negatively correlated with stress_E (*P* = 0.026, *R* = −0.112). There is no linear association between arousal and stress_E. In fact, from Table 2, it can be seen that the degree of stress of the elderly for any photo type is almost the same. Concern is moderately negatively correlated with stress_Y (*p* < 0.001, *R* = −0.465) and has a strong positive correlation with nature (*p* < 0.001, *R* = 0.525). Therefore, pleasure and concern can represent the other indicators in the PAS and NCS scales. In addition, nature is strongly negatively correlated with stress_Y (*p* < 0.001, *R* = −0.729). It is suggested that an increase in stress makes young people talk less naturally and fluently. This is in agreement with the study (Iwamoto et al., 2017), which indicated that young volunteers feel discomfort depending on the type of photo. Young people spoke less when they were emotionally frustrated during times of stress. On the other hand, we found that when young people's concern about the topic increases, the stress of young people decreases. Therefore, by choosing the right type of photo, interest in the topic of the young people increases, thus making the conversation less stressful and more fluent and natural.

### Experiment Evaluation Form Analysis

Details of experiment evaluation form are shown in the Appendix. Q1: The answer of appropriate length of conversation was for all (mean, 1.14; SD, 0.32), for men (mean, 1.25; SD, 0.42), and for women (mean, 1; SD, 0), supporting that the overall mean value of 1 min per photo for conversation is appropriate. Q2: The answer of enjoyment of conversation using photos as topics was for all (mean, 3.18; SD, 0.75), for men (mean, 2.8; SD, 0.75), and for women (mean, 3.6; SD, 0.54). The mean value indicates that the participants were receptive to this format and enjoyed it.

For the answers for Q1, the length of conversation considered appropriate for men was slightly longer than for women. It is not clear if this is due to the higher UCLA mean of 36.83 for men compared with 34.8 for women. The length of conversation using reminiscence therapy for the elderly with different levels of loneliness can be further explored in future studies. For the answers for Q2, the level of pleasure was higher in women than in men. It is not clear whether this is due to the higher GDS mean of 2.6 for women compared with 1.5 for. The effect of reminiscence therapy on individuals with different levels of depression can be further investigated in future studies.

### Annotating Photos of an Old Photo Library by Theme and PC Value

Based on the results in section Overall Analysis and section Single-factor Analysis, it is evident to us that there is a significant difference between concern at the a, b, and ab levels (*P* = 0.001, 0.013, and 0.001) and a significant difference between pleasure at the a level (*P* = 0.006). There was a difference in these two responses to the different photo types in the conversation between the two generations. Meanwhile, according to the results of section Correlation Analysis between Emotion Evaluation Values, the pleasure and concern can represent the other indicators in the evaluation scales. Therefore, the values of pleasure and concern (PC value) were chosen to quantify the conversation effects of the photos in the old photo library. The themes (Showa era: 1, landscape: 2, food: 3, festival: 4) and numbers of the photos were also added to the name. This makes it possible to make a prediction of the effect the photos will have on the conversation when selecting photos for a conversation between two generations. If the photos have different PC values after using the photos multiple times in conversation, the PC values are then averaged and rounded to relabel the photos. There are currently a total of 206 photos in the old photo library. This experiment had a total of 122 photos with positive PC values. The four themes contained 94, 10, 14, and 4 photos, respectively. The detailed distribution is shown in Table 7. With the conversation that had numerous times, the PC values will be continuously averaged. The PC values of the photos will represent the majority group, making the PC values credible and referable enough to be generalized to a wider population.

**Table 7:** Distribution of positive PC values and theme photos.

**PC values**	***C* = 2**	***C* = 3**	***C* = 4**
*P* = 1	3 (1-3)	0	0
*P* = 2	19 (1-15,2-4)	10 (1-9,3-1)	10 (1-7,3-3)
*P* = 3	18 (1-14,3-3,4-1)	17 (1-12,2-2,3-1,4-1)	19 (1-13,2-2,3-3,4-1)
*P* = 4	4 (1-3)	5 (1-5)	17 (1-12,2-1,3-3,4-1)

*1: Showa era, 2: landscape, 3: food, 4: festival; The number after “-” represents the number of the photos*.

## Conclusion

A two-factor experiment was designed to take into account the factors that may affect both sides of the conversation when selecting conversation photos for reminiscence therapy. The emotional state of the conversation for the older people and young people in response to reminiscence therapy was compared using six types of photos. The results of our experimental analysis suggest that:

1. The main factor that influenced pleasure in the conversation among older people was whether they wanted to or not wanted to talk when they saw a photo. But the pleasure value of the conversation was almost always >0 regardless of whether the older people wanted to or did not want to talk. This indicates that the stimulation of memory in the form of photos has a positive effect on the older people, but the pleasantness of older people was enhanced when they saw a photo they wanted to talk about, and the frequency of the pleasure of the conversation reached the highest level.
2. Concern of young people for the topic had a role in the pleasure of the older people in conversation, with a moderately positive correlation linearly. As the concern of the young people for the topic increased, the pleasure for the older people in the conversation also increased. Also, as concern of the young people for the topic increased, stress value of the young people decreased, and the naturalness and fluency of the conversation improved. There was a significant difference in concern of young people for the topic, depending on the six photo types. Especially at the a2b3 level (the older adult does not want to talk about the photo; the young person does not know the topic and is uninterested in the content of the photo), the concern level was lowest. This provides some referential significance for selecting photos that are suitable for a conversation between the two generations; we should avoid choosing these types of photos.
3. Due to the fact that the pleasure level of the conversation for the older people and the concern level of the young people for the topic can reflect other assessment values in PAS and NCS scales, the PC values (pleasure and concern values) provide foresight before the conversation. This value and the theme information (which play the role of finding trigger words when selecting photos) were chosen to quantify the photos. Through this, a library of old photos suitable for the conversation between the two generations was developed. By quantifying photos with PC values and topic information, we hope we can achieve the purpose of stimulating memories of older people and helping young people to easily choose the topics they are interested in, thereby lessening the stress of the young people when talking and increase the pleasure of the older people in conversation.

The limitation of this study is the relatively small research group. Although concern of the young people for the topic had a moderately positive correlation with the pleasure of the older people when conversing, it is not direct evidence (causality), and more data are necessary to support this. Also, not considered was whether PC values differed, depending on gender, education, personality, and other factors. This might mean that PC values may be representative of only a partial group. It is possible that the photo library needs to be used with a larger group to continuously average the PC values in order to minimize the effect of these factors. Another limitation is that the Showa theme occupies the majority of the photo library, and the photo resources of the other themes have yet to be expanded. Perhaps, artificial intelligence technology can learn the characteristics of these photos to help us select more photos for the different themes.

Next, we will make the photo library available to a wider group and integrate the photo library with other communication support systems. Since the level of artificial intelligence technology is limited at this stage, using a remotely operated robotic system for conversation seems to be a more effective and practical solution than communicating and interacting directly with a robot. For example, by using Amazon simple notification service to push a library of old photos to the display of the robot to help older people and volunteers who have never met and communicated with one another. It enables them to have direct conversations (peer to peer) about the content of the photos through the remote bot. In this way, a lot more data can be obtained, and the statistical patterns and case comparisons can be used to pinpoint the right photos for users. Finally, artificial intelligence technology could also be used in terms of automatic recommendations for selecting and adapting photos to improve conversations.

## Data Availability Statement

The original contributions presented in the study are included in the article/Supplementary Material, further inquiries can be directed to the corresponding author/s.

## Ethics Statement

The experiments related to this study were approved by the 116th and 122nd Kyoto Institute of Technology Ethics Committee for Scientific Research Involving Human Subjects (No.2020-18, and 2021-03). The patients/participants provided their written informed consent to participate in this study.

## Author Contributions

LJ and NK designed the experiments. LJ carried out the experiments. LJ and PS analyzed the results. LJ and DC prepared the manuscript. All authors contributed to the article and approved the submitted version.

## Conflict of Interest

The authors declare that the research was conducted in the absence of any commercial or financial relationships that could be construed as a potential conflict of interest.

## Publisher's Note

All claims expressed in this article are solely those of the authors and do not necessarily represent those of their affiliated organizations, or those of the publisher, the editors and the reviewers. Any product that may be evaluated in this article, or claim that may be made by its manufacturer, is not guaranteed or endorsed by the publisher.

## Footnotes

^1^Annual Report on Health and Welfare, Japan's population trends (2013 version) [Online]. The Ministry of Health, Labor, and Welfare. Available: https://www.mhlw.go.jp/wp/hakusyo/kousei/13-2/dl/01.pdf [Accessed 3 February 2021].

^2^White Paper on Population Aging (Section 3 of Chapter 1). [Online]. Cabinet Office. Available: https://www8.cao.go.jp/kourei/whitepaper/w-2019/zenbun/01pdf_index.html [Accessed 8 February 2021].

^3^Mental health of older adults [Online]. World Health Organization. Available: https://www.who.int/news-room/fact-sheets/detail/mental-health-of-older-adults [Accessed 3 February 2021].

^4^*Showa Mono Encyclopedia* [Online]. The Showa Era Lifestyle Museum. Available: https://www.city.kitanagoya.lg.jp/rekimin/mono_index.html [Accessed 10 January 2021].

## Appendix

1. The emotional assessment form, PAS, which was filled out by the older people after the conversation, contained three questions:

Q1. Was the conversation happy? (nine intervals rating from –4 to 4, unhappiness to happiness) Q2. Was the conversation exciting? (nine intervals rating from –4 to 4, clam to exciting,) Q3. Did you feel stressed during the conversation? (levels from 1 to 7, the higher the level, the stronger the pressure)

2. The emotional evaluation form, NCS, which was filled out by the young people after the conversation, contained three questions:

Q1. Did the conversation proceed naturally? (nine intervals rating from –4 to 4, the natural and fluent level of conversation) Q2. Were you interested in the topic during the conversation? (nine intervals rating from –4 to 4, the level of interest in the topic) Q3. Did you feel any stress during the conversation? (levels from 1 to 7, the higher the level, the stronger the pressure)

3. Questions included in the experiment evaluation form that the older people filled out after the conversation:

Q1. Do you think it is appropriate to have a 1-min conversation about the photo? How long do you think is appropriate? (0, not more than 1 min; 1, 1 min; 1.5, 1 and 1/2 min; 2, 2 min or more) Q2. Would you be happy if a young volunteer talked with you regularly about the photos? (4, very happy; 3, happy; 2, somewhat happy; 1, not happy).
